# Respiratory Health Effects of Airborne Particulate Matter: The Role of Particle Size, Composition, and Oxidative Potential—The RAPTES Project

**DOI:** 10.1289/ehp.1104389

**Published:** 2012-05-02

**Authors:** Maciej Strak, Nicole A.H Janssen, Krystal J Godri, Ilse Gosens, Ian S Mudway, Flemming R Cassee, Erik Lebret, Frank J Kelly, Roy M Harrison, Bert Brunekreef, Maaike Steenhof, Gerard Hoek

**Affiliations:** 1 National Institute for Public Health and the Environment (RIVM), Bilthoven, Netherlands; 2 Institute for Risk Assessment Sciences (IRAS), Utrecht University, Utrecht, Netherlands; 3 MRC-HPA Centre for Environmental Health, King’s College London, London, United Kingdom; 4 Division of Environmental Health and Risk Management, University of Birmingham, Birmingham, United Kingdom; 5 Center of Excellence in Environmental Studies, King Abdulaziz University, Jeddah, Saudi Arabia; 6 Julius Centre for Health Sciences and Primary Care, University Medical Centre Utrecht, Utrecht, Netherlands

**Keywords:** air pollution, experimental exposure, FE_NO_, FEV_1_, FVC, oxidative potential, PM, ultrafine particles

## Abstract

Background: Specific characteristics of particulate matter (PM) responsible for associations with respiratory health observed in epidemiological studies are not well established. High correlations among, and differential measurement errors of, individual components contribute to this uncertainty.

Objectives: We investigated which characteristics of PM have the most consistent associations with acute changes in respiratory function in healthy volunteers.

Methods: We used a semiexperimental design to accurately assess exposure. We increased exposure contrast and reduced correlations among PM characteristics by exposing volunteers at five different locations: an underground train station, two traffic sites, a farm, and an urban background site. Each of the 31 participants was exposed for 5 hr while exercising intermittently, three to seven times at different locations during March–October 2009. We measured PM_10_, PM_2.5_, particle number concentrations (PNC), absorbance, elemental/organic carbon, trace metals, secondary inorganic components, endotoxin content, gaseous pollutants, and PM oxidative potential. Lung function [FEV_1_ (forced expiratory volume in 1 sec), FVC (forced vital capacity), FEF_25–75_ (forced expiratory flow at 25–75% of vital capacity), and PEF (peak expiratory flow)] and fractional exhaled nitric oxide (FE_NO_) were measured before and at three time points after exposure. Data were analyzed with mixed linear regression.

Results: An interquartile increase in PNC (33,000 particles/cm^3^) was associated with an 11% [95% confidence interval (CI): 5, 17%] and 12% (95% CI: 6, 17%) FE_NO_ increase over baseline immediately and at 2 hr postexposure, respectively. A 7% (95% CI: 0.5, 14%) increase persisted until the following morning. These associations were robust and insensitive to adjustment for other pollutants. Similarly consistent associations were seen between FVC and FEV_1_ with PNC, NO_2_ (nitrogen dioxide), and NO_x_ (nitrogen oxides).

Conclusions: Changes in PNC, NO_2_, and NO_x_ were associated with evidence of acute airway inflammation (i.e., FE_NO_) and impaired lung function. PM mass concentration and PM_10_ oxidative potential were not predictive of the observed acute responses.

Positive associations between airborne particulate matter (PM) and respiratory health have been observed in epidemiological studies (Brunekreef and Holgate 2002; Pope and Dockery 2006). In most studies, effects were linked to PM_10_ and PM_2.5_ (particulate matter < 10 µm and 2.5 µm in aerodynamic diameter, respectively). Fewer studies have reported health effects associated with exposure to coarse (PM_2.5–10_; Brunekreef and Forsberg 2005) and ultrafine (PM_0.1_; Ibald-Mulli et al. 2002) particles. Depending on sources, there is a significant heterogeneity in PM composition, which is reflected in *in vitro* and *in vivo* toxicological studies (Valavanidis et al. 2008). Current knowledge does not allow precise quantification of the health effects of individual PM components or of PM emissions from different sources [Brunekreef 2010; World Health Organization (WHO) 2007], although various PM characteristics, such as surface area of particles, transition metal content, surface absorbed organic components, and biological products (endotoxin), have been proposed. A measure of oxidative potential (OP) of PM has gained attention as a more integrative measure of biological response (Ayres et al. 2008). OP is an attractive measure because it integrates the effects of multiple individual PM components on health. There is currently, however, very limited evidence in epidemiological studies that the OP of PM predicts health effects better than individual components (Ayres et al. 2008).

Disentangling the independent health effects of individual PM characteristics in epidemiological studies is often limited by high correlations between air pollution components (Brunekreef 2010). Different degrees of measurement error for different air pollution components related to characterizing exposure at a central monitoring location is another problem because more consistent associations tend to be found with air pollution components with less measurement error (Zeger et al. 2000). Controlled experimental exposure studies in laboratory settings cannot wholly represent the complexity of ambient PM exposures and are largely restricted to individual air pollutants or defined mixtures (e.g., diesel engine exhaust). Moreover, experimental concentrations are often higher than those encountered in real-world situations, and the concentration levels used are constant rather than (highly) variable.

Building on recommendations of a recent WHO workshop (WHO 2007), we addressed these uncertainties using a semiexperimental design as part of the RAPTES project (Risk of Airborne Particles: a Toxicological–Epidemiological hybrid Study). We studied health effects of short-term exposure of healthy volunteers to ambient PM at real-world locations with well-established differences in PM characteristics (Strak et al. 2011). The aim of the study was to assess the independent contribution of specific PM characteristics to various health outcomes. Here, we focus on acute changes in respiratory health parameters. We hypothesized that PM_10_ OP would have a stronger and more consistent relationship with airway inflammation and lung function than other measured PM characteristics because oxidative stress is an important mechanism of PM health effects.

## Methods

*Study design.* We exposed healthy human volunteers to ambient PM at five locations with different PM characteristics. A detailed characterization of PM air pollution was performed on-site. Preexposure and postexposure measurements were made to assess respiratory health.

We used a semiexperimental rather than a pure observational design to reduce exposure measurement error. Our design with multiple sampling days at multiple locations used temporal and spatial variability to increase contrast in the measured PM characteristics and reduce correlations. Briefly, we selected five locations in the Netherlands with different source characteristics to increase exposure contrasts and reduce correlations between PM characteristics (Strak et al. 2011). The locations were an underground train station, a continuous traffic location, a stop-and-go traffic location, a farm, and an urban background site. None of the locations were > 70 km away from the collection point located at the Utrecht University campus where all preexposure and some postexposure health measurements were made.

We aimed to recruit 32 volunteers, each undertaking 7 study visits. Each participant had to visit all five locations once, with the two remaining visits assigned randomly to a location. We planned 30 sampling days during March through October 2009, one site per day, with 8 participants exposed during each visit. Results of a previous screening phase showed much higher concentrations at the underground site compared with the outdoor sites, which also strongly influenced correlations between specific air pollutants (Strak et al. 2011). In order to separate the health associations at the underground site and the four outdoor sites, we scheduled 9 visits at the underground and 21 at the remaining four sites. Due to practical constraints, we finally included 31 volunteers, who were measured for an average of 5.5 sampling days (range, 3–7 days). Twenty-six participants visited the underground site at least once, and 13 visited all five sites at least once. In summary, we obtained 45 observations at the underground site and 28–37 at the other sites.

To avoid potential carryover effects from previous exposures, an individual’s visits to the sites were separated by ≥ 14 days. Exposure of each participant was started between 0900 and 0930 hours and lasted for 5 hr. The participants cycled for 20 min on a stationary bicycle each hour. We selected a 5-hr exposure period with intermittent exercise in order to increase the contrast between preexposure and postexposure to ambient air pollution. Moreover, we hypothesized that longer exposure would result in a clearer health response required to study the independent associations with different pollutants. To keep the dose similar among the participants, before the study began we determined for each participant the heart rate corresponding to a minute ventilation rate of 20 L/min/body surface area (square meters) (Zuurbier et al. 2009); we then instructed participants to monitor their heart rates as they cycled in order to maintain the desired ventilation rate. Exercise may affect the measured respiratory variables, but with each participant cycling at a consistent minute ventilation rate each sampling day, bias in associations between the fluctuating air pollution and health end points is likely small. We measured lung function [i.e., forced vital capacity (FVC), forced expiratory volume in 1 sec (FEV_1_), forced expiratory flow between 25% and 75% of FVC (FEF_25–75_), peak expiratory flow (PEF)], measured the fraction of nitric oxide in exhaled air (FE_NO_) as an indicator of airway inflammation, and recorded respiratory symptoms of the participants—all respiratory health indicators that are widely used. These health parameters were measured before exposure (*t* = 0; collection point), before exposure (*t* = 2; sampling location), immediately after exposure (*t* = 7; sampling location), 2 hr after exposure (*t* = 9; collection point), and the next morning (*t* = 25; collection point). The measurements at *t* = 2 were performed to investigate the effect of transport between the collection point and the sampling location. To avoid day-of-week effects, we obtained all measurements on Monday through Thursday of each week.

*Study population.* We recruited 31 healthy, young, nonsmoking participants from among Utrecht University students living on campus to minimize exposure to traffic-related air pollution when traveling to the collection point.

The participants completed an online screening questionnaire. Exclusion criteria included smoking or living in a household with a smoker; lifetime diagnosis of asthma or chronic obstructive pulmonary disease; or history of cardiovascular disease, diabetes mellitus, or pregnancy. Before the study, each participant was examined by a physician and obtained medical clearance for participation.

The study was approved by the ethics committee at University Medical Center Utrecht. Written informed consent was provided by all participants.

*Exposure measurements.* The methods for measuring air pollution on-site during each day of participants’ exposure have been described elsewhere [Strak et al. 2011; see also Supplemental Material, p. 2 (http://dx.doi.org/10.1289/ehp.1104389)]. Briefly, we measured PM_10_ and PM_2.5_, and we determined the absorbance of PM_2.5_ samples and endotoxin content of PM_10_ samples. We made real-time measurements of particle number concentration (PNC) and the gaseous pollutants ozone (O_3_), nitrogen dioxide (NO_2_), and (nitrogen oxides (NO_x_). We measured the concentrations of elemental carbon (EC), organic carbon (OC), trace metals, nitrate, and sulfate in PM_2.5–10_ and PM_2.5_ samples. OP was measured in three fractions—PM_2.5–10_, PM_0.18–2.5_, and PM_0.18_—and assessed *in vitro* by measuring antioxidant depletion of ascorbate (OP^AA^) and reduced glutathione (OP^GSH^) (Godri et al. 2010).

We equipped a minibus with a custom-made cabin air filter to minimize exposure during transport of participants between the collection point and the sampling locations.To estimate traffic-related air pollution during transport, we measured the PNC in the minibus during each commute.

*Clinical measurements.* FE_NO_ was measured with a Niox Mino monitor (Aerocrine, Solna, Sweden), an instrument that complies with recommendations from the American Thoracic Society/European Respiratory Society (ATS/ERS 2005). The instrument was also used to measure ambient-air nitric oxide levels where FE_NO_ measurements were performed.

Lung function parameters (FVC, FEV_1_, FEF_25–75_, PEF) were measured with an EasyOne electronic spirometer (ndd, Zürich, Switzerland), which meets ATS/ERS spirometry standards (Miller et al. 2005). Each participant performed at least three maneuvers supervised by one of the eight technicians operating the device. The best value from the technically correct maneuvers was selected according to the maximum value method of the European Respiratory Society (Quanjer et al. 1993). After each sampling day, two syringe checks using a calibrated 3-L syringe were recorded to monitor the accuracy of the device, which had to be within 3%.

At each time point, a short questionnaire was administered to subjectively grade respiratory symptoms that were present (e.g., cough, congestion/rhinorrhea, wheeze): symptoms were scaled from 0 (no complaints) to 3 (severe complaints).

Before the morning health measurements, the participants completed a questionnaire reporting additional exposure to traffic- or workplace-related air pollution, medication use, and so forth during the preceding 24 hr.

*Data analyses.* We analyzed the associations between air pollution concentrations during exposure at the sampling locations and the difference in lung function and FE_NO_ between postexposure (*t* = 7, *t* = 9, *t* = 25) and preexposure (*t* = 0) for each sampling day using mixed linear regression. We used mixed models to account for the influence of repeated observations per participant (using compound symmetry of the residuals). We did not include sampling location in the analysis because it was not significantly associated with the outcome after including exposure and potential confounders. We used the 5-hr average concentrations of air pollutants measured at the locations as independent variables. For OP and trace metals, the data from the individual PM size fractions were aggregated. We first specified single-pollutant models. Then, to study the individual associations of different pollutants, we specified two-pollutant models with all possible combinations of measured pollutants. Here, we primarily report and discuss the results of the two-pollutant models. Models in which two pollutants had a Spearman’s rank correlation coefficient (*r*_S_) > 0.7 were considered highly correlated and were not interpreted. We defined a large number of models, so we focused on the consistency of significant associations and not on single isolated significant associations. Because there was a substantial difference in some exposure parameters between outdoor locations and the underground location, we also analyzed the data separately for the outdoor locations and the underground location. To offset potential confounders, we adjusted for temperature and relative humidity measured at the location during sampling, the season in which the sampling day occurred (before or after the start date of the calendar summer), an indicator variable for low/high grasses and birch pollen counts, and an indicator variable for respiratory infection. Pollen counts were obtained from the station in Leiden, located within 50 km of our sampling locations. We selected grass and birch pollen as highly allergenic indicator pollen, with good spatial correlation with pollen counts at another station. Because the distribution was highly skewed, we included pollen as a low/high variable (Brunekreef et al. 2000). For the lung function measurements, we investigated possible technician and instrument effects, but neither had an effect.

Sensitivity analysis assessed participants who *a*) did not report nasal allergies, *b*) were not former smokers, and *c*) did not take anti-inflammatory medication in the 24-hr period before the start of the sampling day. We assessed the impact of influential values on the regression results by comparing effect estimates with and without the 1% of observations with the highest Cook’s distance value.

Effect estimates and their confidence intervals (CI) are presented as percentage increases over a study population mean of the baseline (*t* = 0) values. We express these values as percentage increases per change in interquartile ranges (IQR) for the outdoor locations. We calculated IQRs for endotoxin concentrations for all locations, but without the levels measured at the farm location. We used these IQRs in the analysis of the complete data set and the outdoor-only data set to allow direct comparison of effect estimates. Statistical significance was defined as *p* < 0.05 and borderline significance as *p* < 0.10. All data analyses were carried out using SAS, version 9.2 (SAS Institute Inc., Cary, NC, USA).

## Results

A total of 170 observations were obtained from 31 participants (Table 1) who were exposed at least three and at most seven times. Each participant visited the underground train station at least once. We did not analyze the data from the respiratory symptoms questionnaires because only questions about congestion/rhinorrhea and cough reported > 15% changes in scores over the sampling days.

**Table 1:** Population characteristics and baseline (t = 0) FENO and lung function.

Characteristic	Value
Age (years)	22 (19–26)
Sex [n (%)]	
Female	21 (68)
Male	10 (32)
Nasal allergy [n (%)]a	5 (16)
Former smoker [n (%)]	3 (10)
Body mass index (kg/m2)	22.3 (17.0–32.0)
FENO (ppb)b	15.9 (5–61)
FEV1 (L)c	3.86 (2.57–5.51)
FVC (L)c	4.68 (2.73–6.70)
FEF25–75 (L/sec)c	3.94 (2.06–6.43)
PEF (L/sec)c	8.71 (5.38–14.68)
Unless otherwise stated, values are mean (range). aIncludes hay fever. bN = 151–169. cN = 165–170.	

*Exposure measurements.* The measurements at the underground train station showed substantially higher concentrations of nearly every PM characteristic, especially levels of coarse PM, iron (Fe), copper (Cu) and the sum of OP^AA^ and OP^GSH^ (OP^TOTAL^) [see Supplemental Material, Table S1 (http://dx.doi.org/10.1289/ehp.1104389)]. For example, the mean concentration of coarse PM was 252 µg/m^3^ at the station and 13 µg/m^3^ at the outdoor sites. PNC was the highest at the continuous traffic site at 66,500 particles/cm^3^, and substantially increased levels of endotoxin were measured at the farm. Variability of concentrations was large at the outdoor sites but more limited within the underground-only data set. Therefore, we will not report underground-only air pollution effect estimates.

Correlations between air pollution concentrations are shown in Table 2. PM_10_ and PM_2.5_ were highly correlated with each other, as well as with absorbance, EC, OC, trace metals, and OP^TOTAL^, but not with PNC. The high correlations decreased considerably after we excluded the measurements from the underground train station. However, as a result of the exclusion, we observed a substantial increase in correlations between PNC and EC or absorbance; the correlations between PNC and PM_10_ or PM_2.5_ remained. O_3_ showed a strong negative correlation with several PM characteristics. Overall, the correlations between several PM characteristics were sufficiently low to investigate their independent associations with health end points.

**Table 2:** Spearman’s rank correlation coefficients (rS) between PM characteristics.

PM_10_	PM_2.5_	PM_2.5–10_	PNC	Abs^a^	EC(C)	EC(F)	OC(C)	OC(F)	Fe(tot)	Fe(sol)	Cu(tot)	Cu(sol)	Ni(tot)	Ni(sol)	V(tot)	V(sol)	Endo	NO_3_^–a^	SO_4_^2–a^	OP^AA^	OP^GSH^	OP^TOTAL^	O_3_	NO_2_	NO_x_
PM10		0.94	0.82	0.22	0.74	0.70	0.69	0.76	0.73	0.70	0.44	0.70	0.80	0.76	0.00	0.66	–0.25	0.36	0.07	–0.15	0.88	0.82	0.89	–0.67	0.26	0.37
PM2.5	0.88		0.67	0.15	0.68	0.66	0.64	0.68	0.79	0.62	0.43	0.65	0.74	0.71	–0.04	0.67	–0.26	0.34	0.18	–0.04	0.91	0.79	0.88	–0.65	0.21	0.31
PM2.5–10	0.55	0.22		0.21	0.71	0.68	0.67	0.78	0.46	0.70	0.46	0.69	0.73	0.79	0.01	0.58	–0.27	0.37	–0.29	–0.44	0.73	0.79	0.77	–0.65	0.14	0.35
PNC	0.19	0.07	0.15		0.65	0.60	0.67	0.00	–0.04	0.62	0.65	0.60	0.56	0.07	0.47	0.25	0.17	–0.32	–0.27	–0.16	0.36	0.32	0.35	–0.37	0.50	0.70
Absa	0.37	0.22	0.31	0.84		0.88	0.98	0.48	0.49	0.92	0.75	0.89	0.92	0.64	0.19	0.66	–0.19	–0.01	–0.30	–0.36	0.80	0.73	0.78	–0.81	0.39	0.70
EC(C)	0.28	0.17	0.26	0.77	0.73		0.89	0.45	0.36	0.88	0.75	0.92	0.93	0.54	0.25	0.77	–0.06	–0.07	–0.24	–0.41	0.79	0.77	0.80	–0.71	0.27	0.61
EC(F)	0.25	0.13	0.19	0.86	0.96	0.77		0.42	0.43	0.92	0.74	0.89	0.90	0.60	0.24	0.71	–0.15	–0.02	–0.35	–0.38	0.79	0.73	0.76	–0.81	0.36	0.67
OC(C)	0.52	0.39	0.57	–0.06	0.00	–0.04	–0.13		0.46	0.49	0.27	0.54	0.57	0.62	–0.08	0.43	–0.25	0.59	–0.07	–0.22	0.70	0.77	0.77	–0.48	–0.01	0.15
OC(F)	0.59	0.72	0.06	–0.20	0.05	–0.26	–0.07	0.08		0.37	0.20	0.37	0.53	0.62	–0.18	0.37	–0.35	0.22	0.36	0.08	0.66	0.48	0.60	–0.50	0.19	0.21
Fe(tot)	0.24	0.04	0.27	0.90	0.83	0.77	0.81	0.07	–0.22		0.78	0.96	0.88	0.57	0.15	0.62	–0.26	–0.07	–0.31	–0.55	0.72	0.69	0.72	–0.67	0.27	0.54
Fe(sol)	–0.05	–0.11	–0.01	0.86	0.65	0.59	0.66	–0.23	–0.27	0.80		0.79	0.74	0.40	0.31	0.48	–0.07	–0.15	–0.38	–0.44	0.51	0.43	0.45	–0.51	0.08	0.56
Cu(tot)	0.28	0.12	0.26	0.82	0.76	0.82	0.77	0.12	–0.23	0.93	0.69		0.92	0.53	0.24	0.67	–0.17	–0.05	–0.24	–0.46	0.76	0.75	0.76	–0.70	0.24	0.57
Cu(sol)	0.55	0.41	0.37	0.71	0.85	0.83	0.80	0.15	0.09	0.78	0.55	0.82		0.63	0.19	0.66	–0.16	0.00	–0.14	–0.31	0.82	0.80	0.83	–0.73	0.34	0.64
Ni(tot)	0.40	0.27	0.49	–0.09	0.11	–0.11	–0.01	0.22	0.37	–0.10	–0.11	–0.16	0.13		0.07	0.67	–0.20	0.40	–0.10	–0.24	0.72	0.68	0.73	–0.67	0.11	0.24
Ni(sol)	–0.01	–0.06	0.00	0.46	0.35	0.43	0.46	–0.22	–0.37	0.27	0.49	0.36	0.26	0.11		0.44	0.74	0.06	0.03	0.30	0.11	0.11	0.04	–0.37	0.26	0.37
V(tot)	0.14	0.19	–0.05	0.20	0.19	0.47	0.29	–0.18	–0.18	0.04	0.06	0.21	0.22	0.16	0.75		0.20	0.27	–0.11	–0.16	0.79	0.70	0.76	–0.80	0.16	0.35
V(sol)	0.04	0.07	0.00	0.19	0.14	0.42	0.24	–0.17	–0.30	0.00	0.11	0.13	0.13	0.16	0.81	0.96		–0.07	0.15	0.51	–0.15	–0.22	–0.20	–0.01	0.23	0.21
Endo	0.22	0.22	0.22	–0.37	–0.30	–0.49	–0.31	0.40	0.13	–0.52	–0.45	–0.49	–0.42	0.20	0.08	0.05	0.14		–0.05	0.10	0.27	0.38	0.30	–0.32	–0.15	–0.19
NO3–a	0.56	0.74	–0.10	–0.26	–0.12	–0.05	–0.21	0.11	0.64	–0.22	–0.27	–0.09	0.11	0.18	–0.13	0.20	0.06	0.02		0.67	0.03	–0.19	–0.05	0.17	0.27	–0.17
SO42–a	0.50	0.72	–0.12	–0.14	0.08	–0.07	0.05	0.12	0.54	–0.32	–0.29	–0.19	0.10	0.33	0.20	0.49	0.39	0.33	0.66		–0.10	–0.22	–0.16	0.12	0.29	–0.07
OPAA	0.75	0.79	0.32	0.28	0.51	0.47	0.45	0.40	0.38	0.26	0.03	0.35	0.56	0.24	0.07	0.47	0.36	0.00	0.57	0.67		0.82	0.95	–0.78	0.32	0.47
OPGSH	0.54	0.48	0.44	0.12	0.27	0.39	0.28	0.50	–0.01	0.14	–0.15	0.35	0.47	0.15	0.02	0.21	0.15	0.13	0.10	0.34	0.54		0.92	–0.69	0.14	0.35
OPTOTAL	0.73	0.73	0.40	0.22	0.42	0.50	0.35	0.56	0.24	0.23	–0.11	0.38	0.59	0.28	–0.07	0.37	0.26	–0.02	0.42	0.54	0.88	0.80		–0.72	0.28	0.41
O3	–0.21	–0.15	–0.18	–0.35	–0.57	–0.33	–0.57	0.07	–0.06	–0.18	–0.14	–0.26	–0.35	–0.20	–0.54	–0.52	–0.48	–0.21	–0.03	–0.47	–0.46	–0.21	–0.27		–0.36	–0.57
NO2	0.49	0.45	0.28	0.56	0.74	0.60	0.67	0.06	0.26	0.52	0.34	0.52	0.71	0.28	0.28	0.36	0.27	–0.19	0.26	0.32	0.69	0.28	0.58	–0.62		0.65
NOx	0.32	0.21	0.25	0.75	0.87	0.71	0.87	–0.11	0.01	0.70	0.53	0.66	0.72	0.14	0.47	0.39	0.35	–0.23	–0.05	0.13	0.54	0.21	0.42	–0.68	0.91	
Abbreviations: Abs, absorbance; C, coarse PM fraction; Endo, endotoxin; F, fine PM fraction; sol, water-soluble metal extraction; tot, total. Values in the light-blue area represent correlations in the outdoor-only data set. aMeasured in PM2.5.																										

*Associations between air pollution and FE_NO._* Single-pollutant models. We observed significant associations with a range of pollutants including PNC, NO_x_, absorbance, fine-fraction (F) EC [EC(F)], OC(F), OP, Fe, Cu, vanadium (V), and water-soluble nickel (Ni) [see Supplemental Material, p. 3 (http://dx.doi.org/10.1289/ehp.1104389); see also Strak et al. 2012, Table R1.] [Tables in the web-based additional supplement (Strak et al. 2012) are denoted with an “R” to distinguish them from tables in the Supplemental Material (denoted with “S”); information presented in the web-based additional supplement has not been peer reviewed.]

Two-pollutant models. Immediately after exposure, the associations for PNC at 11.2% (95% CI: 5.5, 17.0%) remained unchanged after adjustment for all other pollutants (Table 3; PNC row) and cancelled out the other significant association of EC(F) seen in the univariate model (Table 3; PNC column). For the outdoor locations, PNC and Fe showed the most consistent associations in the two-pollutant models [see Supplemental Material, Table S2 (http://dx.doi.org/10.1289/ehp.1104389)]. We could not study individual associations with PNC and Fe because they were highly correlated.

**Table 3:** Two-pollutant models of associations between air pollution exposure and percentage changes (postexposure – preexposure) in FENO immediately after exposure (all sites).

IQR	Adjustment pollutant														
	PM_10_	PM_2.5_	PM_2.5–10_	PNC	Abs^a^	EC(F)	EC(C)	OC(F)	OC(C)	Fe(tot)	Fe(sol)	Cu(tot)	Cu(sol)	Ni(tot)	Ni(sol)
			V(tot)	V(sol)	Endo	NO3–a	SO42–a	OPAA	OPGSH	OPTOTAL	O3	NO2	NOx
PM10	13.50	0.09	0.68	–1.00	–0.02	–1.27**	–1.35**	0.40	0.28	0.22	0.73	0.09	0.93	0.23	0.58	0.07			PM10	0.28	0.08	0.00	0.10	0.06	0.56	0.54	0.69	–0.11	0.00	–0.08
PM2.5	11.54	–1.43	0.17	–0.86	–0.02	–2.43**	–2.64**	0.10	0.57	0.38	–0.03	0.15	1.03	0.44	0.73	0.12			PM2.5	0.34	0.15	–0.03	0.25	0.12	0.98	0.42	0.83	–0.42	–0.05	–0.27
PM2.5–10	8.23	1.02	0.41	0.10	–0.02	–1.17**	–1.23**	0.41	0.28	0.22	0.99	0.09	0.80	0.23	0.63	0.08			PM2.5–10	0.30	0.08	0.01	0.09	0.06	0.52	0.68	0.70	–0.04	0.01	–0.06
PNC	32,906	11.28**	11.26**	11.30**	11.24**	11.80**	11.44**	11.28**	11.57**	11.07**	11.17**	11.56**	11.23**	11.30**	11.06**	11.30**			PNC	11.17**	10.94**	10.93**	11.55**	11.02**	14.66**	14.58**	14.63**	12.04**	14.66**	14.85**
Absa	3.49	10.74**	8.76**	10.68**	–0.55	2.41	–13.81	11.51**	4.45**	3.42*	12.39**	2.90	11.21**	3.95**	7.07**	2.37			Absa	6.60**	2.27	1.69	2.16	2.09	10.10**	10.51**	11.53**	9.58**	1.62	1.73
EC(F)	4.35	12.75**	10.61**	12.58**	–0.21	18.26	2.92*	11.80**	4.87**	4.19**	15.33**	3.36*	13.09**	4.32**	8.84**	2.88			EC(F)	7.91**	2.76	2.18	2.63	2.54	11.16**	10.39**	11.85**	9.96**	2.12	2.35
EC(C)	0.40	–0.41	0.07	–0.46	–0.11	–1.91**	–1.70**	0.12	0.42	0.18	–0.03	0.08	0.88	0.38	0.45	0.07			EC(C)	0.17	0.10	–0.04	0.11	0.07	0.71	0.46	0.79	–0.15	0.00	–0.17
OC(F)	1.82	–2.81	–2.48	–2.91	–1.92	–4.58*	–4.23*	–2.90	–1.10	–1.04	–2.44	–2.38	–2.31	–2.05	–1.72	–1.24			OC(F)	–1.68	–0.66	–2.21	–1.04	–1.10	–2.88	–4.11	–3.63	–2.77	–1.98	–2.37
OC(C)	0.79	–0.79	–0.53	–0.88	0.28	–1.42	–1.58	–0.38	0.33	0.12	–0.48	–0.02	–0.18	0.25	–0.03	–0.13			OC(C)	–0.20	–0.30	0.06	0.52	0.00	1.28	1.37	1.37	–0.40	–0.01	–0.35
Fe(tot)	895.10	–0.10	0.02	–0.15	–0.01	–0.24**	–0.26**	0.02	0.04	0.02	0.01	0.01	0.18	0.03	0.08	0.01			Fe(tot)	0.03	0.01	0.00	0.01	0.01	0.07	0.05	0.08	–0.02	0.00	–0.01
Fe(sol)	32.09	0.18	0.25	0.15	–0.66	–0.68	–0.62	0.22	1.21	0.41	0.23	0.40	0.39	1.39	0.42	0.25			Fe(sol)	0.33	0.41	–0.18	0.37	0.32	0.66	0.36	0.49	0.14	0.07	–0.33
Cu(tot)	57.96	–0.18	–0.08	–0.17	–0.02	–0.29**	–0.30**	–0.12	0.05	0.02	–0.24	0.00	0.01	0.03	0.04	0.00			Cu(tot)	0.00	0.01	–0.01	0.01	0.00	0.06	0.00	0.04	–0.04	–0.01	–0.03
Cu(sol)	8.65	–0.05	–0.04	–0.05	–0.04	–0.10	–0.09	–0.07	0.04	–0.01	–0.04	–0.07	–0.04	0.00	–0.02	–0.02			Cu(sol)	–0.02	–0.01	–0.04	0.00	0.00	–0.01	–0.04	–0.02	–0.04	–0.02	–0.06
Ni(tot)	3.53	–0.71	–0.33	–0.83	–0.02	–1.11**	–1.25**	–0.37	0.21	0.05	–0.67	–0.01	–0.18	0.11	0.05	–0.01			Ni(tot)	–0.10	0.02	–0.08	–0.01	–0.06	0.34	0.15	0.27	–0.23	–0.03	–0.16
Ni(sol)	1.82	1.05	1.15	1.00	–0.79	–0.06	–0.08	1.14	1.71	1.43	1.12	1.18	1.32	1.59	1.38	1.34			Ni(sol)	1.54	0.06	0.92	1.09	0.64	1.29	1.21	1.26	0.95	0.50	0.52
V(tot)	2.04	–0.38	–0.12	–0.47	–0.11	–1.45*	–1.55**	–0.09	0.33	0.18	–0.27	0.07	0.15	0.21	0.25	–0.06			V(tot)	0.13	0.00	–0.05	0.11	0.03	0.11	–0.08	0.03	–0.35	–0.06	–0.14
V(sol)	1.94	2.65	2.70	2.62	2.22	2.44	2.38	2.74	2.73	3.15	2.71	2.86	2.78	2.95	2.82	2.79			V(sol)	2.84	2.84	2.75	4.09	3.14	2.47	2.35	2.42	2.54	1.67	1.98
Endo	0.19	–0.07	–0.07	–0.07	–0.01	–0.05	–0.05	–0.07	–0.08*	–0.07	–0.07	–0.07	–0.07	–0.08	–0.07	–0.06			Endo	–0.07	–0.07	–0.07	–0.07	–0.07	–0.07	–0.06	–0.07	–0.07	–0.05	–0.05
NO3–a	5.19	–2.12	–2.21	–2.08	0.46	–1.68	–1.59	–2.09	–2.09	–2.31	–2.06	–2.09	–2.09	–2.11	–2.12	–2.03			NO3–a	–2.09	–2.66	–1.93	–2.11	–1.07	–4.08*	–4.13*	–4.10*	–2.17	–2.19	–1.89
SO42–a	2.99	–1.99	–2.02	–1.97	–0.44	–1.54	–1.43	–2.09	–2.05	–2.15	–2.09	–2.12	–2.14	–2.15	–2.23	–2.04			SO42–a	–2.13	–2.26	–1.94	–1.32	–2.05	–2.86	–2.83	–2.84	–2.06	–2.26	–1.91
OPAA	19.08	–0.16	–0.12	–0.16	–0.03	–0.38**	–0.37**	–0.15	0.08	–0.04	–0.13	–0.02	–0.07	0.02	–0.06	0.00			OPAA	0.00	0.01	–0.02	0.01	–0.01	0.01	–0.09	–0.19	–0.07	–0.02	–0.05
OPGSH	15.53	–0.12	–0.03	–0.17	–0.02	–0.31**	–0.27**	–0.07	0.10	–0.03	–0.06	0.00	0.01	0.04	–0.01	0.00			OPGSH	0.02	0.01	–0.01	0.02	0.01	0.08	0.01	0.15	–0.03	–0.01	–0.03
OPTOTAL	38.71	–0.20	–0.09	–0.22	–0.03	–0.45**	–0.40**	–0.17	0.11	–0.04	–0.14	–0.01	–0.04	0.04	–0.04	0.00			OPTOTAL	0.01	0.01	–0.02	0.02	0.00	0.20	–0.18	0.01	–0.07	–0.01	–0.05
O3	9.74	–2.16	–2.72	–1.64	1.56	10.61**	9.52**	–2.12	–2.90	–1.61	–2.16	–1.09	–2.96	–2.06	–2.47	–0.90			O3	–2.65	–0.88	–0.04	–1.37	–1.25	–2.88	–2.15	–2.71	–1.23	0.92	1.40
NO2	10.54	6.87	6.98	6.81	–7.40	4.42	4.12	6.54	7.94*	6.53	6.49	6.46	6.71	6.87	6.62	6.28			NO2	6.71	5.93	4.89	7.03	7.33	6.56	6.43	6.51	8.02	6.88	5.03
NOx	28.05	5.31	5.49	5.18	–5.77	2.00	1.52	5.49	5.95*	4.65	5.00	4.87	5.58	6.23	5.15	4.19			NOx	4.82	3.98	2.95	4.33	4.40	5.82	5.47	5.68	6.13	1.63	4.65
Abbreviations: Abs, absorbance; C, coarse PM fraction; Endo, endotoxin; F, fine PM fraction; sol, water-soluble metal extraction; tot, total. Values in light blue boxes indicate rS > 0.7. Values in each row represent effect estimates for the pollutants in two-pollutant models; values in dark blue boxes forming a diagonal are effect estimates in a single-pollutant model. All models were adjusted for temperature, relative humidity, season, pollen counts, respiratory infection, and adjustment pollutant. Estimates are percentage increases above population-average baseline expressed per IQR of outdoor-sites (N = 170), except for all models including OP (N = 153) and all models including EC(C), OC(C), and trace metals (N = 166). aMeasured in PM2.5. *p < 0.10. **p < 0.05.																														

Findings for 2 hr after exposure were very similar (see Strak et al. 2012, Table R4). PNC was most consistently associated with FE_NO_ in the full data set. In the outdoor-only models, the consistent significant associations of PNC, coarse-fraction (C) EC [EC(C)], water-soluble Cu, and total Fe could not be further disentangled (see Strak et al. 2012, Table R5).

The morning after exposure, associations were weaker than at previous time points (see Strak et al. 2012, Table R6). The most consistent associations were found for water-soluble Ni and V, which remained similar after adjusting for all the other pollutants. However, those associations were driven by one single influential observation and decreased and became nonsignificant after excluding this observation. The water-soluble fractions of Ni and V were too highly correlated to study their individual associations with FE_NO_. PNC associations at 7.3% (95% CI: 0.5, 14.1%) were less stable than at the first two time points, with modestly reduced nonsignificant effect estimates after adjusting for water-soluble Ni and NO_x_. The associations with water-soluble Ni and V were not present in the outdoor-only data set, even though their concentrations were not increased at the underground location (see Strak et al. 2012, Table R7).

*Associations between air pollution and lung function.* Single-pollutant models. We observed significant associations of FVC and FEV_1_ with a range of pollutants including NO_x_, PNC, absorbance, EC, Fe, Cu, and water-soluble Ni. None of the exposure parameters were associated with changes in PEF and FEF_25–75_ at any time point [see Supplemental Material, pp. 3–4 (http://dx.doi.org/10.1289/ehp.1104389); see also Strak et al. 2012, Tables R2, R3].

Two-pollutant models. Immediately after exposure, fairly consistent associations with FVC were found for PNC, NO_2_, and water-soluble Ni (Table 4). In the outdoor-only models, we observed the most consistent associations for PNC and NO_x_, respectively, at –1.3% (95% CI: –2.4, –0.3%) and –2.4% (95% CI: –3.8, –1.0%), which decreased and became nonsignificant after adjusting for O_3_ (and PNC after adjusting for NO_2_) [see Supplemental Material, Table S4 (http://dx.doi.org/10.1289/ehp.1104389)]. A positive association with O_3_ in the single-pollutant models was consistent. For FEV_1_, the only consistent pattern was a positive association with OC(F) in the complete data set (see Strak et al. 2012, Tables R12–R13).

**Table 4:** Two-pollutant models of associations between air pollution exposure and percentage changes (postexposure – preexposure) in FVC immediately after exposure (all sites).

IQR	Adjustment pollutant														
	PM_10_	PM_2.5_	PM_2.5–10_	PNC	Abs^a^	EC(F)	EC(C)	OC(F)	OC(C)	Fe(tot)	Fe(sol)	Cu(tot)	Cu(sol)	Ni(tot)	Ni(sol)
		V(tot)	V(sol)	Endo	NO3–a	SO42–a	OPAA	OPGSH	OPTOTAL	O3	NO2	NOx
PM10	13.50	0.02	–0.34*	0.70**	0.03	0.20**	0.18**	–0.16	–0.02	0.05	0.17	0.02	0.04	0.00	–0.05	0.05			PM10	0.13*	0.03	0.02	0.02	0.02	–0.08	0.00	–0.07	0.26**	0.06	0.07*
PM2.5	11.54	0.90**	0.08	0.60**	0.11	0.50**	0.47**	0.15	0.00	0.17	0.57*	0.09	0.47	0.07	0.11	0.16*			PM2.5	0.39**	0.10	0.09	0.08	0.08	–0.01	0.20	0.11	0.64**	0.17*	0.22**
PM2.5–10	8.23	–0.64**	–0.21*	0.01	0.03	0.14*	0.12	–0.20*	–0.03	0.03	–0.13	0.01	–0.06	–0.01	–0.10	0.04			PM2.5–10	0.08	0.02	0.02	0.01	0.01	–0.10	–0.06	–0.11	0.21**	0.04	0.06
PNC	32,906	–1.26**	–1.26**	–1.26**	–1.19**	–1.47**	–1.47**	–1.34**	–1.26**	–1.19**	–1.27**	–1.31**	–1.27**	–1.25**	–1.22**	–1.01*			PNC	–1.19**	–1.18**	–1.31**	–1.41**	–1.32**	–1.40**	–1.36**	–1.39**	–1.15**	–0.60	–1.15*
Absa	3.49	–1.37**	–1.39**	–1.11*	0.27	–0.10	–1.10	–1.84**	–0.45	–0.10	–1.38**	–0.19	–1.15*	–0.32	–0.83*	0.10			Absa	–0.11	–0.09	–0.08	–0.11	–0.13	–1.13**	–0.82	–1.13**	0.62	0.32	0.47
EC(F)	4.35	–1.36**	–1.46**	–1.06	0.31	1.12	–0.10	–1.56**	–0.43	–0.09	–1.41*	–0.17	–1.11*	–0.28	–0.89*	0.12			EC(F)	–0.08	–0.07	–0.07	–0.11	–0.14	–1.08**	–0.66	–0.98*	0.64	0.37	0.52
EC(C)	0.40	0.24	–0.04	0.32*	0.07	0.37**	0.28**	0.04	–0.02	0.08	0.30	0.05	0.32	0.04	0.03	0.09*			EC(C)	0.16*	0.04	0.05	0.04	0.04	–0.02	0.17	0.07	0.33**	0.09*	0.14**
OC(F)	1.82	0.61	0.50	0.67*	0.55*	0.79**	0.73**	0.55	0.50	0.52	0.63	0.67*	0.67	0.73	0.51	0.67**			OC(F)	0.71**	0.46	0.58*	0.50	0.50	0.56	0.79*	0.68	0.85**	0.72**	0.71**
OC(C)	0.79	–0.24	–0.32	–0.18	–0.05	0.00	–0.01	–0.24	–0.12	–0.04	–0.17	–0.06	–0.18	–0.16	–0.27	0.10			OC(C)	–0.01	0.01	–0.04	–0.04	–0.05	–0.55**	–0.69**	–0.64**	0.09	0.00	0.06
Fe(tot)	895.10	–0.02	–0.03*	0.02	0.00	0.03**	0.03*	–0.03	0.00	0.01	0.00	0.00	–0.01	0.00	–0.01	0.01			Fe(tot)	0.02	0.00	0.00	0.00	0.00	–0.02	0.00	–0.01	0.03**	0.01	0.01
Fe(sol)	32.09	0.00	–0.06	0.02	0.16	0.12	0.10	–0.08	–0.18	0.06	0.01	0.04	–0.01	–0.14	–0.03	0.15			Fe(sol)	0.07	0.03	0.06	0.04	0.04	–0.13	0.00	–0.06	0.16	0.14	0.23
Cu(tot)	57.96	–0.01	–0.04	0.02	0.01	0.04*	0.03*	–0.05	–0.01	0.01	0.01	0.00	0.00	0.00	–0.01	0.01			Cu(tot)	0.02	0.00	0.00	0.00	0.00	–0.02	0.00	–0.02	0.04**	0.01	0.02*
Cu(sol)	8.65	0.01	0.00	0.01	0.01	0.01	0.01	0.00	–0.01	0.01	0.01	0.01	0.01	0.01	0.00	0.01			Cu(sol)	0.01	0.01	0.01	0.01	0.01	0.00	0.00	0.00	0.02	0.01	0.02*
Ni(tot)	3.53	0.10	–0.02	0.18	0.05	0.18**	0.17*	0.01	–0.01	0.09	0.16	0.04	0.12	0.03	0.04	0.09*			Ni(tot)	0.16*	0.04	0.04	0.04	0.04	–0.08	0.01	–0.05	0.20**	0.07	0.10*
Ni(sol)	1.82	–0.98**	–1.02**	–0.95**	–0.62*	–0.83**	–0.83**	–1.01**	–0.98**	–0.83**	–0.99**	–0.86**	–1.02**	–0.95**	–1.01**	–0.77**			Ni(sol)	–1.29**	–0.73	–0.78**	–0.78**	–0.82**	–0.93**	–1.02**	–0.98**	–0.74*	–0.54	–0.66*
V(tot)	2.04	–0.24	–0.29**	–0.17	0.00	0.00	0.00	–0.21*	–0.10	–0.02	–0.24	–0.03	–0.19	–0.06	–0.21*	0.14			V(tot)	–0.02	0.02	–0.02	–0.02	–0.02	–0.16	–0.15	–0.17	0.14	0.05	0.03
V(sol)	1.94	–0.56	–0.59	–0.55	–0.53	–0.52	–0.52	–0.55	–0.52	–0.53	–0.55	–0.53	–0.55	–0.54	–0.56	–0.07			V(sol)	–0.58	–0.53	–0.53	–0.54	–0.52	–1.34**	–1.40**	–1.37**	–0.47	–0.18	–0.43
Endo	0.19	0.00	0.00	0.00	0.00	0.00	0.00	0.00	0.01	0.00	0.00	0.00	0.00	0.00	0.00	0.00			Endo	0.00	0.00	0.00	0.00	0.00	0.00	0.00	0.00	0.00	–0.01	0.00
NO3–a	5.19	–0.06	–0.08	–0.05	–0.35	–0.08	–0.08	–0.04	–0.07	–0.04	–0.04	–0.05	–0.04	–0.07	–0.01	–0.09			NO3–a	–0.05	0.03	–0.06	–0.06	0.06	–0.19	–0.18	–0.19	–0.05	–0.02	–0.10
SO42–a	2.99	–0.10	–0.09	–0.10	–0.28	–0.15	–0.15	–0.07	–0.13	–0.11	–0.08	–0.10	–0.08	–0.10	–0.05	–0.20			SO42–a	–0.11	–0.05	–0.11	–0.16	–0.12	–0.07	–0.08	–0.07	–0.12	–0.03	–0.13
OPAA	19.08	0.03	0.01	0.04	0.01	0.05**	0.05**	0.02	0.00	0.03**	0.04	0.02	0.04	0.01	0.03	0.02			OPAA	0.03*	0.01	0.01	0.01	0.01	0.01	0.04	0.08	0.06**	0.02*	0.03**
OPGSH	15.53	0.00	–0.02	0.02	0.01	0.03	0.02	–0.03	–0.01	0.03**	0.01	0.00	0.00	0.00	0.00	0.01			OPGSH	0.02	0.01	0.01	0.01	0.00	–0.03	0.00	–0.06	0.04**	0.01	0.02
OPTOTAL	38.71	0.03	0.00	0.05	0.01	0.05**	0.04*	–0.01	–0.01	0.03**	0.03	0.01	0.03	0.01	0.02	0.02			OPTOTAL	0.03	0.01	0.01	0.01	0.01	–0.07	0.09	0.01	0.06**	0.02*	0.02*
O3	9.74	2.58**	2.53**	2.24**	0.07	1.08	1.01	2.32**	0.80**	0.43	2.20**	0.49	1.68**	0.70*	1.38**	0.07			O3	0.91	0.25	0.34	0.34	0.34	1.78**	1.61**	1.86**	0.34	–0.27	–0.07
NO2	10.54	–2.19**	–2.27**	–2.13**	–1.26	–2.31**	–2.30**	–2.22**	–2.25**	–1.81**	–2.13**	–1.93**	–2.11**	–1.99**	–2.05**	–1.50**			NO2	–2.02**	–1.67**	–2.06**	–1.81**	–1.80**	–2.33**	–2.27**	–2.32**	–2.17**	–1.82**	–2.31**
NOx	28.05	–1.50**	–1.63**	–1.40**	–0.06	–1.64**	–1.61**	–1.86**	–1.27**	–0.92	–1.44**	–1.22**	–1.53**	–1.52**	–1.38**	–0.63			NOx	–0.99*	–0.77	–1.01*	–0.91*	–0.90*	–1.51**	–1.34**	–1.45**	–0.97	0.47	–0.89*
Abbreviations: Abs, absorbance; C, coarse PM fraction; Endo, endotoxin; F, fine PM fraction; sol, water-soluble metal extraction; tot, total. Values in light blue boxes indicate rS > 0.7. Values in each row represent effect estimates for the pollutants in two-pollutant models; values in dark blue boxes forming a diagonal are effect estimates in a single-pollutant model. All models were adjusted for temperature, relative humidity, season, pollen counts, respiratory infection, and adjustment pollutant. Estimates are percentage increases above population-average baseline expressed per IQR of outdoor-sites (N = 170), except for all models including OP (N = 153) and all models including EC(C), OC(C), and trace metals (N = 166). aMeasured in PM2.5. *p < 0.10, **p < 0.05.																														

Two hours after exposure, the most consistent associations for FVC were found with NO_2_ and NO_x_, respectively, at –1.5% (95% CI: –2.8, –0.3%) and –1.1% (95% CI: –2.1, –0.1%), although the latter decreased somewhat after adjusting for PNC (see Strak et al. 2012, Table R8). We did not interpret models with NO_2_ and NO_x_ further because NO_2_ is a part of NO_x_. In the outdoor-only models, the significant associations with NO_x_ disappeared after adjusting for O_3_ and EC(C) (see Strak et al. 2012, Table R9). The positive association with O_3_ was weaker after adjusting for NO_x_. The association of NO_2_ with FEV_1_ at –1.6% (95% CI: –2.8, –0.3%) observed in single-pollutant models remained stronger than other associations in two-pollutant models (see Strak et al. 2012, Table R14). In the outdoor-only data set, NO_x_ was most consistently associated with FEV_1_ (see Strak et al. 2012, Table R15).

The morning after exposure, NO_2_ and NO_x_ were consistently associated with a drop in FVC (see Strak et al. 2012, Table R10) with the effect estimates of –1.9% (95% CI: –3.2, –0.6%) and –1.4% (95% CI: –2.4, –0.4%), respectively. In the outdoor models, the associations of NO_x_ with FVC were insensitive to adjusting for other pollutants except for O_3_ (see Strak et al. 2012, Table R11), for which there was a fairly consistent positive association. For FEV_1_, NO_x_ had a fairly consistent negative association (see Strak et al. 2012, Table R16), whereas sulfate had a fairly consistent positive association. In the outdoor data set, the fairly consistent association with NO_x_ remained (see Strak et al. 2012, Table R17) with an effect estimate of –1.3% (96% CI: –2.5, –0.2%).

*Additional analyses.* Exposure of participants to PNC during transport to and from the sampling sites was not associated with changes in respiratory health and did not affect associations with the experimental exposures. Pollen counts were the only variables not measured on-site. Analyses with and without pollen counts resulted in similar associations.

Exclusion of the three former smokers and the five participants with reported nasal allergy showed similar associations between air pollutants and FE_NO_, FVC, and FEV_1_. Exclusion of the 12 observations with anti-inflammatory medication did not change the effect estimates.

## Discussion

We investigated acute respiratory health effects in a panel of healthy, young volunteers semiexperimentally exposed to ambient air pollution with contrasting PM characteristics. We found associations of PNC, NO_2_, NO_x_, absorbance, EC, and trace metals with changes in FE_NO_, FVC, and FEV_1_ immediately after, 2 hr after, and the morning after exposure. The most consistent associations in two-pollutant models were between PNC and FE_NO_ and between NO_2_/NO_x_ and lung function. Changes in those parameters were not consistently related to PM mass concentration, sulfate/nitrate content, or OP of particles.

We used a semiexperimental design to study the independent associations between respiratory function and a large number of PM characteristics. That design allowed us to define two-pollutant models to investigate independent associations of single pollutants with fewer problems than observational studies. Because we observed participants in a semiexperimental setting, exposure measurement error was largely due to instrumental errors; therefore, issues such as representativeness of outdoor central monitoring for personal exposure do not affect our study. Instrumental precision of measurements was between 5% and 10%, which is very low compared with the range of measured concentrations. Furthermore, PNC and NO_x_ were not more precisely measured than the other components [with the possible exception of total Cu and Ni; see Supplemental Material, Table S3 (http://dx.doi.org/10.1289/ehp.1104389)]. Difference in instrumental precision is thus an unlikely explanation for their stronger associations. Correlations between PM characteristics were reduced by performing repeated measurements at multiple locations with different source characteristics, although some correlations remained too high to interpret in two-pollutant models.

In the present study, we observed a consistent association between PNC and increased FE_NO_ immediately after and 2 hr after exposure. This association was insensitive to adjustment by any of the 25 other pollutants. PNC was not highly correlated with other pollutants in the whole data set. In the outdoor-only models, we could not disentangle the associations with PNC, absorbance, EC, Fe, and Cu because they were highly correlated. However, the latter components were dominant at the underground site but their associations were insignificant in the data set including underground, and the PNC association was consistently strong in both the whole data set and the outdoor-only data set, thus suggesting that the associations with absorbance, EC, Fe, and Cu for the outdoor sites are likely explained by those with PNC.

PNC especially reflects the ultrafine particles for which respiratory health effects have been documented in previous epidemiological and controlled exposure studies (Ibald-Mulli et al. 2002). The consistency of PNC effects is notable when we take into account the low correlation of FE_NO_ with FVC and FEV_1_. Our results for lung function are contrary to findings of two experimental studies with higher PNC concentrations than in our study (Larsson et al. 2007; Samet et al. 2009). The shorter duration of these studies (2 hr) may explain the lack of a lung function response. In a study in Arnhem, the Netherlands, Zuurbier et al. (2011) reported a 5% increase in FE_NO_ in response to an 18,000-particles/cm^3^ (IQR) increase in PNC measured 6 hr after a 2-hr exposure in a car or bus. This increase corresponds to a 9% increase in FE_NO_ if expressed per our IQRs, roughly comparable with our findings. Similarly, Strak et al. (2009) reported a 13% increase in FE_NO_ in cyclists after a 1-hr commute, as expressed per the IQRs in our study. McCreanor et al. (2007) reported that a 2-hr walk near heavy diesel traffic resulted in an approximately 4% increase in FE_NO_ expressed per our IQR.

Similarly consistent associations were observed between NO_2_ and NO_x_ and lung function parameters 2 hr after and the morning after the exposure. PNC was most consistently related to FVC immediately after exposure. There is still a debate whether the associations observed between respiratory health and NO_2_ at the concentrations currently found in western European countries are due to direct effects of NO_2_ or other PM components co-varying with NO_2_ (WHO 2006). In the present study, we measured a detailed set of PM characteristics including PNC as a proxy for ultrafine particles and metals, but we still observed associations with NO_2_.

For some components that were higher at the underground station than at other locations, we observed associations only in the outdoor-only models. This likely argues against a causal role, although saturation of biological parameters after exposure to very high air pollution concentrations could also provide an explanation.

Exposure to PM mass, irrespective of the size fraction, was not associated with acute respiratory health changes. The lack of association between PM_10_ and PM_2.5_ and acute changes in respiratory function is consistent with the results from two Dutch studies on bicycle commuting (Strak et al. 2009; Zuurbier et al. 2011) in which increases in FE_NO_ were not associated with mass-based PM metrics but PNC and absorbance were.

In contrast to our hypothesis, OP did not display a strong and consistent relationship with acute respiratory health effects. In the single-pollutant models, associations of OP metrics with increased FE_NO_ 2 hr after exposure were evident in the outdoor-only models, whereas they disappeared in the two-pollutant models. In our primary analyses, we used the OP of aggregated PM fractions. A secondary investigation of OP of the smallest available fraction (PM_0.18_) did not show consistent associations with the measured respiratory parameters, nor did it reduce the associations observed for PNC and NO_x_. Our characterization of OP focused on PM and did not include oxidative properties of other co-pollutants, such as O_3_ and NO_2_. However, in the two-pollutant models with those co-pollutants, we did not observe consistent associations with OP. Our measurement of AA or GSH depletion is one of the existing methods of PM OP determination, and it is possible that other methods would show associations that we did not observe. The assay we used examined only the intrinsic potential of PM to drive oxidation reactions in an acellular model—reflecting its redox-active transition metals and quinone content. Upon interaction with airway cells, PM can elicit oxidative stress through alternative pathways; therefore, this assay can account for only a fraction of *in vivo* PM activity.

## Conclusions

Changes in PNC, NO_2_, and NO_x_ were associated with evidence of acute airway inflammation (FE_NO_) and impaired lung function. These associations were robust and insensitive to adjustment for other pollutants. PM mass concentration and PM_10_ OP were not predictive of the observed acute responses.

## Supplemental Material

(131 KB) PDF

## References

[r1] ATS/ERS (American Thoracic Society, European Respiratory Society) (2005). ATS/ERS recommendations for standardized procedures for the online and offline measurement of exhaled lower respiratory nitric oxide and nasal nitric oxide, 2005.. Am J Respir Crit Care Med.

[r2] Ayres JG, Borm P, Cassee FR, Castranova V, Donaldson K, Ghio A (2008). Evaluating the toxicity of airborne particulate matter and nanoparticles by measuring oxidative stress potential—a workshop report and consensus statement.. Inhal Toxicol.

[r3] Brunekreef B (2010). The color of smoke.. Epidemiology.

[r4] Brunekreef B, Forsberg B (2005). Epidemiological evidence of effects of coarse airborne particles on health.. Eur Respir J.

[r5] Brunekreef B, Hoek G, Fischer P, Spieksma FTM (2000). Relation between airborne pollen concentrations and daily cardiovascular and respiratory-disease mortality.. Lancet.

[r6] Brunekreef B, Holgate ST (2002). Air pollution and health.. Lancet.

[r7] Godri KJ, Duggan ST, Fuller GW, Baker T, Green D, Kelly FJ (2010). Particulate matter oxidative potential from waste transfer station activity.. Environ Health Perspect.

[r8] Ibald-Mulli A, Wichmann H-E, Kreyling W, Peters A (2002). Epidemiological evidence on health effects of ultrafine particles.. J Aerosol Med.

[r9] Larsson B-M, Sehlstedt M, Grunewald J, Sköld CM, Lundin A, Blomberg A (2007). Road tunnel air pollution induces bronchoalveolar inflammation in healthy subjects.. Eur Respir J.

[r10] McCreanor J, Cullinan P, Nieuwenhuijsen MJ, Stewart-Evans J, Malliarou E, Jarup L (2007). Respiratory effects of exposure to diesel traffic in persons with asthma.. N Engl J Med.

[r11] Miller MR, Hankinson J, Brusasco V, Burgos F, Casaburi R, Coates A (2005). Standardisation of spirometry.. Eur Respir J.

[r12] Pope CA, Dockery DW (2006). Health effects of fine particulate air pollution: lines that connect.. J Air Waste Manag Assoc.

[r13] Quanjer PH, Tammeling GJ, Cotes JE, Pedersen OF, Peslin R, Yernault JC (1993). Lung volumes and forced ventilatory flows. Report Working Party Standardization of Lung Function Tests, European Community for Steel and Coal. Official statement of the European Respiratory Society.. Eur Respir J Suppl.

[r14] Samet JM, Rappold A, Graff D, Cascio WE, Berntsen JH, Huang Y-CT (2009). Concentrated ambient ultrafine particle exposure induces cardiac changes in young healthy volunteers.. Am J Respir Crit Care Med.

[r15] Strak M, Boogaard H, Meliefste K, Oldenwening M, Zuurbier M, Brunekreef B (2009). Respiratory health effects of ultrafine and fine particle exposure in cyclists.. Occup Environ Med.

[r16] Strak M, Janssen NA, Godri KJ, Gosens I, Mudway IS, Cassee FR (2012). Additional Supplementary Material: Respiratory Health Effects of Airborne Particulate Matter: The Role of Particle Size, Composition and Oxidative Potential - The RAPTES Project.. http://www.raptes.nl/rsc/pdf/strak-2012-raptes-ehp.pdf.

[r17] Strak M, Steenhof M, Godri KJ, Gosens I, Mudway IS, Cassee FR (2011). Variation in characteristics of ambient particulate matter at eight locations in the Netherlands - the RAPTES project.. Atmos Environ.

[r18] Valavanidis A, Fiotakis K, Vlachogianni T (2008). Airborne particulate matter and human health: toxicological assessment and importance of size and composition of particles for oxidative damage and carcinogenic mechanisms.. J Environ Sci Health C Environ Carcinog Ecotoxicol Rev.

[r19] WHO (World Health Organization) (2006). Air Quality Guidelines. Global Update 2005. Particulate Matter, Ozone, Nitrogen Dioxide, and Sulfur Dioxide. Copenhagen:WHO Regional Office for Europe.. http://www.euro.who.int/__data/assets/pdf_file/0005/78638/E90038.pdf.

[r20] WHO (World Health Organization) (2007). Health Relevance of Particulate Matter from Various Sources.. http://www.euro.who.int/__data/assets/pdf_file/0007/78658/E90672.pdf.

[r21] Zeger SL, Thomas D, Dominici F, Samet JM, Schwartz J, Dockery D (2000). Exposure measurement error in time-series studies of air pollution: concepts and consequences.. Environ Health Perspect.

[r22] Zuurbier M, Hoek G, Oldenwening M, Meliefste K, van den Hazel P, Brunekreef B (2011). Respiratory effects of commuters’ exposure to air pollution in traffic.. Epidemiology.

[r23] Zuurbier M Hoek G van den Hazel P Brunekreef B 2009 Minute ventilation of cyclists, car and bus passengers: an experimental study. Environ Health 8 48 doi:10.1186/1476-069X-8-48 [Online 27 October 2009] 19860870 PMC2772854

