# Supplementary material for: Respiratory Health Effects of Airborne Particulate Matter: The Role of Particle Size, Composition, and Oxidative Potential—The RAPTES Project
Source: Environ Health Perspect. 2012 May 2;120(8):1183–9. doi: 10.1289/ehp.1104389 (PMC3440077; doi:10.1289/ehp.1104389)
Supplement: (131 KB) PDF [file ehp.1104389.s001.pdf]

SUPPLEMENTAL MATERIAL

Respiratory Health Effects of Airborne Particulate Matter: The Role of Particle Size, Composition and Oxidative Potential - The RAPTES Project

Maciej Strak, Nicole A.H. Janssen, Krystal J. Godri, Ilse Gosens, Ian S. Mudway, Flemming R. Cassee,
Erik Lebret, Frank J. Kelly, Roy M. Harrison, Bert Brunekreef, Maaike Steenhof, Gerard Hoek

Supplement text Pages 2-4

Table S1 Geometric means and minimum-maximum of 5-hour average air pollution concentrations Page 5

Table S2 Two-pollutant models of associations between air pollution exposure and percentage changes (post-pre) in FE_{NO} immediately after exposure (outdoor sites) Page 6

Table S3 Precision and limits of detection (LOD) for 5-hour sampling periods Page 7

Table S4 Two-pollutant models of associations between air pollution exposure and percentage changes (post-pre) in FVC immediately after exposure (outdoor sites) Page 8

Exposure measurements

We determined gravimetrically concentrations of PM_{10} and $PM_{2.5}$, and measured the absorbance of those samples using a smoke stain reflectometer. We calculated $PM_{2.5\text{-}10}$ mass concentrations as the differences between PM_{10} and $PM_{2.5}$. Endotoxin content of PM_{10} samples was measured using an LAL assay. PNC was measured using a condensation particle counter. With a high volume sampler we collected $PM_{2.5\text{-}10}$ and $PM_{2.5}$ samples in which we measured the concentrations of EC and OC, trace metals, e.g., iron (Fe), copper (Cu), nickel (Ni), vanadium (V) (both water-soluble and "total" acid-extracted fractions), as well as inorganic (nitrate, sulfate) components. Additionally, gaseous pollutants concentrations (O_3, NO, NO_2, NO_X) were measured using real-time monitors (U.V. Photometric O_3 Analyzer model 49; Thermo Environmental Instruments, Franklin, MA, and Chemiluminescence NO/NO_2/NO_X Analyzer model 200E; Teledyne API, San Diego, CA). PM for OP measurement was collected using Micro-Orifice Uniform Deposit cascade Impactor (MSP Corporation, Minneapolis, MN), which sampled PM in three fractions - $PM_{2.5\text{-}10}$, $PM_{0.18\text{-}2.5}$ and $PM_{0.18}$. OP was assessed *in vitro* by measuring antioxidant depletion in a synthetic human respiratory tract lining fluid (RTLF) containing physiologically relevant concentrations (200 µM) of ascorbate (AA) and reduced glutathione (GSH). OP was determined by the extent of AA (OP^{AA}) and GSH (OP^{GSH}) depletion relative to particle-free controls. The percentage depletion, expressed per unit mass, was then calculated per cubic meter of ambient air (Godri et al. 2010). The sum of both metrics is presented as OP^{TOTAL}.

PNC exposure during transport of participants was measured with a portable real-time condensation particle counter (CPC model 3007; TSI, St Paul, MN). The CPC 3007 measures particles ranging above 0.01 µm in diameter. Before each sampling day a "zero" check was performed.

Single-pollutant models of associations between air pollution and FE_{NO}

PNC was positively associated with participants' FE_{NO} immediately after exposure, two hours after exposure, and the next morning (RAPTES 2012; Table R1). EC (F) had a positive association with FE_{NO} immediately after exposure and two hours thereafter. Nitrogen oxides, absorbance, OC (F), V and water-soluble Ni were positively associated with FE_{NO} at single time points. O_3 showed an association with FE_{NO} in an opposite to expected direction two hours after exposure. In the outdoor sites dataset, the associations seen in the whole dataset for PNC remained similar, while absorbance and EC were associated at all time points. Additionally, positive associations of FE_{NO} with Fe and Cu appeared immediately after and two hours after exposure, while positive associations with OP^{GSH} and OP^{TOTAL} appeared two hours after exposure and the following morning. Increase in OP^{AA} and water-soluble Ni was associated with increase in FE_{NO} at single time points. Overall, the effect estimates from the mixed models were comparable between the adjusted and unadjusted model. Deletion of 1% observations with the highest Cook's Distances generally did not affect the effect estimates.

Single-pollutant models of associations between air pollution and lung function

NO_2 and NO_X were associated with decrease in FVC at all time points. PNC was associated with drops in FVC immediately after exposure and the next morning, while water-soluble Ni - immediately after and two hours after the exposure (RAPTES 2012; Table R2). Nitrogen oxides were also associated with drops in FEV_1 two hours after and the morning after the exposure (RAPTES 2012; Table R3). PNC, absorbance, EC (F) and water-soluble Ni were associated with drops in FEV_1 at single time points. We also observed positive associations of FEV_1 with OC (F) (immediately after exposure) and sulfate (two hours after and the morning after exposure). In the outdoor datasets, nitrogen oxides were associated with

drops in FVC and FEV_1 across all time points, and the associations became stronger than observed in the whole dataset. Additionally, PNC, EC and absorbance were also negatively associated with FVC across all time points. At two time points after exposure, Cu was associated with drops in FVC while absorbance was associated with decrease in FEV_1. PNC, EC, Fe, Cu and water-soluble Ni were associated with FVC or FEV_1 at single time points. O_3 showed a positive association with FVC at all time points after exposure, as well as with FEV_1 two hours after exposure, while OC (F) was positively associated with both FVC and FEV_1 immediately after exposure. None of the exposure parameters were associated with changes in PEF and $FEF_{25\text{-}75}$ at any time point. The effect estimates from the mixed models were overall similar between the adjusted and unadjusted models and the deletion of 1% observations with the highest Cook's Distances had generally no influence on the effect estimates.

Table S1 Geometric means and minimum-maximum of 5-hour average air pollution concentrations.

	All sites	Outdoor sites	Underground	Continuous traffic	Stop-and-go traffic	Farm	Urban background
**PM_{10}**	76 (18-450)	37 (18-130)	394 (354-450)	40 (36-44)	34 (21-77)	55 (30-130)	26 (18-37)
**$PM_{2.5}$**	39 (8-167)	23 (8-95)	140 (123-167)	23 (17-39)	20 (13-63)	36 (18-95)	16 (8-30)
**$PM_{2.5-10}$**	32 (4-282)	13 (4-35)	252 (212-282)	13 (4-22)	13 (8-18)	18 (12-35)	9 (7-13)
**PNC**	23.0 (7.0-74.7)	20.7 (7.0-74.7)	29.4 (14.6-39.8)	66.5 (60.0-74.7)	29.4 (12.8-42.6)	9.6 (8.1-11.2)	9.1 (7.0-11.8)
**Absorbance[a]**	4 (0.3-16)	2 (0.3-8)	14 (11-16)	6 (5-8)	3 (1-6)	1 (0.3-3)	1 (1-2)
**EC (F)**	4 (0.3-19)	2 (0.3-7)	15 (12-19)	6 (6-7)	3 (1-6)	1 (0.3-2)	1 (1-2)
**EC (C)**	0.3 (0.0004-10)	0.07 (0.0004-0.5)	8 (6-10)	0.4 (0.4-1)	0.3 (0.2-0.5)	0.02 (0.0004-0.2)	0.01 (0.0004-0.3)
**OC (F)**	2 (0.6-11)	1 (0.6-7)	4 (2-11)	1 (1-4)	1 (1-7)	1 (1-3)	1 (1-2)
**OC (C)**	2 (0.5-6)	1 (0.5-5)	4 (3-6)	1 (1-2)	1 (1-2)	3 (2-5)	1 (0.5-1)
**Fe (tot)**	3,699 (132-176,699)	690 (132-2,655)	154,408 (133,299-176,699)	2,008 (1,287-2,655)	884 (698-1,362)	277 (132-513)	365 (245-486)
**Fe (sol)**	48 (7-431)	33 (7-75)	114 (24-431)	61 (53-75)	40 (24-71)	15 (7-32)	25 (16-43)
**Cu (tot)**	160 (4-8,193)	29 (4-97)	7,001 (5,267-8,193)	90 (75-97)	35 (23-72)	12 (4-35)	16 (13-22)
**Cu (sol)**	24 (2-1,637)	6 (2-18)	517 (189-1,637)	15 (12-18)	6 (3-13)	5 (3-8)	3 (2-7)
**Ni (tot)**	9 (0.5-78)	4 (0.5-31)	68 (59-78)	3 (2-4)	4 (2-8)	7 (0.5-31)	3 (2-22)
**Ni (sol)**	2 (0.6-10)	2 (0.6-5)	2 (0.9-10)	2 (1-5)	2 (1-4)	1 (1-3)	1 (1-3)
**V (tot)**	6 (0.5-49)	3 (0.5-12)	25 (18-49)	3 (2-6)	4 (1-12)	2 (0.5-5)	3 (1-6)
**V (sol)**	2 (0.1-10)	2 (0.3-10)	0.9 (0.1-5)	2 (1-5)	3 (1-10)	1 (0.3-4)	2 (1-4)
**Endotoxin**	1 (0.3-44)	1 (0.3-44)	0.7 (0.6-1)	0.4 (0.3-1)	0.5 (0.3-1)	17 (11-44)	1 (0.3-1)
**NO_3^- [a]**	4 (0.6-39)	5 (1-39)	3 (0.6-9)	3 (1-8)	4 (2-19)	7 (1-39)	5 (3-19)
**SO_4^{2-} [a]**	3 (1-21)	3 (1-21)	2 (1-5)	3 (2-6)	3 (1-5)	4 (2-10)	4 (2-21)
**OP_{AA}**	103 (12-2,527)	27 (12-99)	1,480 (996-2,527)	31 (24-36)	31 (16-99)	30 (14-97)	20 (12-40)
**OP_{GSH}**	79 (4-2,505)	18 (4-45)	1,580 (1,066-2,505)	30 (23-41)	17 (14-19)	24 (7-45)	10 (4-29)
**OP_{TOTAL}**	190 (16-5,032)	47 (16-142)	3,082 (2,368-5,032)	61 (48-76)	50 (31-115)	57 (21-142)	31 (16-69)
**O_3**	7 (0.3-32)	18 (6-32)	0.8 (0.3-6)	15 (6-24)	16 (10-32)	21 (13-26)	22 (16-30)
**NO_2**	20 (9-34)	20 (9-34)	20 (14-26)	23 (22-30)	25 (15-34)	17 (10-26)	14 (9-18)
**NO_X**	36 (14-96)	32 (14-96)	45 (15-69)	49 (43-60)	47 (18-96)	22 (14-34)	19 (14-26)

[a] measured in $PM_{2.5}$.

Absorbance is expressed in 10^{-5}/m; endotoxin is presented in EU/m^3; PM_{10}, $PM_{2.5}$, EC, OC, NO_3^-, SO_4^{2-} are expressed in µg/m^3; PNC in 10^3/cm^3; Fe, Cu, Ni and V in ng/m^3; OP in 1/m^3; O_3, NO_2 and NO_X in ppb. "Tot" denotes total, while "sol" water-soluble metal extraction. "C" is the coarse and "F" is the fine PM fraction.

Table S2 Two-pollutant models of associations between air pollution exposure and percentage changes (post-pre) in FE_{NO} immediately after exposure (outdoor sites).

	IQR	ADJUSTMENT POLLUTANTS																								
		PM_{10}	$PM_{2.5}$	$PM_{2.5-10}$	PNC	Abs.[a]	EC (F)	EC (C)	OC (F)	OC (C)	Fe (tot)	Fe (sol)	Cu (tot)	Cu (sol)	Ni (tot)	Ni (sol)	V (tot)	V (sol)	End.	NO_3^{-} [a]	SO_4^{2-} [a]	OP^{AA}	OP^{GSH}	OP^{TOTAL}	O_3	NO_2	NO_X
PM_{10}	13.50	0.40	11.96	-6.46	1.24	0.95	0.92	-0.70	-1.04	1.87	-0.12	3.12	0.18	0.43	0.29	0.20	-0.48	-0.48	0.88	5.50	1.52	2.38	-2.34	-4.58	0.37	0.17	0.41
$PM_{2.5}$	11.54	-15.75	-0.45	-5.52	2.02	1.43	1.52	-1.27	-2.56	-0.54	0.43	4.77	0.52	0.70	-0.64	-0.05	-2.07	-1.85	0.30	10.27*	1.85	-17.88	-6.21	-14.64*	-0.53	-0.49	-0.01
$PM_{2.5-10}$	8.23	11.22	7.29	2.31	1.11	1.06	0.77	-0.23	0.74	6.15	-1.31	3.31	-0.28	0.37	2.27	0.86	1.49	1.37	2.68	5.40	2.44	9.59*	3.35	7.11	2.33	1.63	1.61
PNC	32,906	11.20**	11.58**	10.85**	11.01**	25.72**	13.26	15.64**	10.34**	11.21**	-16.51	-8.38	-6.88	1.37	11.18**	9.16**	10.60**	10.64**	10.53**	11.50**	10.57**	15.94**	18.84**	17.74**	13.89**	16.66**	18.56**
Absorbance[a]	3.49	12.54**	12.86**	12.18**	-19.50	12.39**	-47.20*	15.36	11.67**	12.81**	-23.93**	-13.67	-14.41	-9.59	12.40**	9.27*	11.71**	11.88**	11.88**	12.18**	11.70**	17.96**	17.87**	18.84**	19.11**	26.97**	31.15**
EC (F)	4.35	15.98**	16.40**	15.65**	-3.45	70.26**	15.85**	23.66**	14.88**	16.14**	-37.01**	-10.26	-19.16	-5.24	15.83**	12.66**	15.11**	15.08**	15.50**	15.91**	15.06**	20.48**	21.45**	21.57**	21.63**	30.22**	36.70**
EC (C)	0.40	13.00**	12.94**	12.74**	-8.99	-4.65	-9.96	12.61**	11.19*	12.61**	-18.27*	-8.03	-18.93*	-13.66	14.39**	8.43	13.20**	12.34**	11.70**	12.88**	13.31**	20.52**	15.21*	21.34**	31.44**	31.39**	29.39**
OC (F)	1.82	-14.40	-15.70*	-12.35	-7.78	-9.92	-8.56	-5.16	-12.92	-15.82*	-4.07	-7.75	-2.45	-5.56	-11.63	-0.05	-11.80	-13.08	-15.58*	-16.45*	-13.55*	-14.61	-11.13	-13.66	-13.53	-12.32	-11.36
OC (C)	0.79	-1.86	0.08	-3.49	1.30	1.50	1.39	-0.14	-2.28	-0.26	0.17	3.35	0.45	0.78	0.02	-0.46	-0.72	-1.00	0.75	1.33	0.02	6.00	-2.74	2.46	-0.31	0.00	0.25
Fe (tot)	895.10	11.14**	11.15**	11.48**	24.49**	25.76**	31.35**	19.41**	10.75**	11.14**	11.12**	4.44	14.79	11.56*	11.47**	10.54**	11.04**	10.98**	11.63**	11.16**	10.80**	13.04**	13.97**	13.86**	12.94**	15.69**	17.57**
Fe (sol)	32.09	21.33**	22.50**	19.75**	30.19**	32.64**	28.02**	24.77**	18.89**	22.22**	13.02	19.41**	16.96*	21.02**	19.80**	19.08**	19.23**	19.32**	21.39**	22.05**	19.27**	27.56**	27.69**	27.78**	23.03**	26.34**	28.76**
Cu (tot)	57.96	15.97**	16.05**	16.07**	24.19**	28.96**	31.47**	29.13**	15.56**	16.07**	-5.64	2.48	15.98**	14.99	17.29**	15.51**	16.07**	15.90**	16.97**	15.89**	15.43**	19.83**	19.86**	20.62**	19.30**	22.90**	26.13**
Cu (sol)	8.65	14.43**	14.54**	14.34**	12.89	22.84**	18.47*	23.63**	13.74**	14.64**	-0.71	-1.61	1.06	14.43**	14.63**	12.77**	14.30**	14.21**	14.21**	14.20**	13.80**	16.85**	18.66**	18.05**	19.09**	23.86**	25.75**
Ni (tot)	3.53	-0.54	-0.57	-0.62	0.41	0.32	0.33	0.69	-0.08	-0.54	0.46	0.33	0.85	0.21	-0.54	-0.45	-0.34	-0.45	-0.40	-0.93	-1.01	0.20	0.76	1.10	-0.78	-0.41	-0.32
Ni (sol)	1.82	12.55**	12.56**	12.31**	4.81	7.35	5.50	8.32	12.54*	12.61**	2.05	0.67	0.99	4.17	12.46**	12.58**	13.97**	17.74**	11.88**	11.82**	11.26*	13.33**	12.61**	13.15**	13.69**	13.02**	13.04**
V (tot)	2.04	3.31	4.36	2.44	1.63	1.64	1.08	-0.96	0.03	3.25	0.76	2.08	-0.27	0.69	2.53	-1.96	2.93	0.74	2.53	6.76	6.79	1.42	-1.15	-1.04	4.27	2.22	1.86
V (sol)	1.94	4.11	5.03	3.05	3.01	3.47	2.49	0.81	-1.12	4.43	1.90	3.49	0.45	1.72	3.41	-6.39	2.86	3.69	3.97	6.24	4.93	3.60	1.25	2.17	4.16	3.06	2.71
Endotoxin	0.19	-0.08	-0.07	-0.08	-0.02	-0.01	-0.01	-0.03	-0.09*	-0.07	0.02	0.05	0.03	-0.01	-0.06	-0.05	-0.06	-0.07	-0.07	-0.07	-0.07	-0.06	-0.11*	-0.07	-0.08	-0.07	-0.06
NO_3^{-} [a]	5.19	-6.04*	-8.46*	-3.76	0.71	-0.33	0.06	-2.33	-3.20	-2.80	0.08	2.25	-0.18	-0.44	-2.68	-1.21	-3.90*	-3.11	-1.92	-2.09	-0.19	-14.59**	-5.92**	-9.35**	-2.44	-2.10	-1.75
SO_4^{2-} [a]	2.99	-2.97	-3.14	-2.58	-0.82	-1.64	-1.29	-3.01	-2.72	-2.62	-0.76	-0.18	-0.86	-1.55	-3.16	-1.42	-4.15**	-2.97	-2.51	-2.41	-2.55	-4.82**	-4.14**	-4.66**	-3.36	-2.70	-2.43
OP^{AA}	19.08	-2.10	15.52	-5.73	-4.50	-4.51	-3.49	-8.20*	-1.74	-4.24	-4.45	-1.87	-5.09	-3.54	0.37	-0.09	-0.67	-0.81	0.30	16.84**	3.76	0.55	-3.07	-10.99	1.63	-1.08	-1.21
OP^{GSH}	15.53	6.43	7.64	2.57	-7.32	-3.15	-3.36	-2.50	1.90	6.27	-4.97	-1.18	-3.74	-4.60	5.09	2.23	4.48	4.01	8.09*	5.96	4.67	6.45	4.59	8.94	5.60	4.08	3.33
OP^{TOTAL}	38.71	9.50	17.39*	-3.10	-8.08	-5.98	-5.03	-10.00	-0.50	0.06	-6.74	-2.23	-6.69	-5.63	5.28	1.24	3.04	1.97	4.41	12.88**	5.75	16.07	-6.22	3.23	5.80	1.92	1.05
O_3	9.74	-0.26	-0.69	0.39	10.61*	14.91**	12.58*	26.42**	1.82	0.97	10.09*	11.28*	11.06*	13.37**	2.97	4.60	4.09	2.30	1.79	-2.91	-5.35	2.56	4.07	5.27	-0.47	14.11	12.14
NO_2	10.54	4.84	4.91	4.11	-13.63*	-23.06**	-20.54**	-22.87**	4.00	3.89	-14.20**	-13.35**	-14.05**	-17.72**	3.39	-1.17	2.63	2.97	2.24	4.93	5.57	5.19	1.96	3.17	16.44	4.90	-22.75
NO_X	28.05	5.46	5.45	5.02	-13.21**	-22.41**	-21.95**	-16.15*	4.09	4.73	-14.27**	-12.97**	-14.58**	-15.94**	4.28	-0.77	3.91	3.98	3.46	4.83	5.14	6.09	3.88	5.06	12.67*	23.10	5.45

[a] measured in $PM_{2.5}$.

*p<0.10, **p<0.05.

"Tot" denotes total, while "sol" water-soluble metal extraction. "C" is the coarse and "F" is the fine PM fraction.

Fields in light shading indicate Spearman's R above 0.7. In each row effect estimates for the indicated pollutant in two-pollutant models are presented. The effect estimates in a single-pollutant model are presented on the diagonal (dark shading). All models were adjusted for temperature, relative humidity, season, pollen counts, respiratory infection and adjustment pollutant (indicated in the header of each column). Estimates are percentage increases above population-average baseline expressed per outdoor-sites IQR. N=170, except all models including OP where N=153 and all models including EC (C), OC (C) and trace metals where N=166.

Table S3 Precision and limits of detection (LOD) for 5-hour sampling periods.

Component	Precision	LOD (% > LOD)
PM_{10}	4.0	9.77 (98%)
$PM_{2.5}$	4.6	9.77 (98%)
Absorbance[a]	4.5	0.58 (95%)
EC	8.4	-
OC	9.5	-
Fe (tot)	2.4	67.79 (100%)
Fe (sol)	6.0	74.26 (79%-90%)
Cu (tot)	15.8	2.07 (100%)
Cu (sol)	5.2	3.03 (93%-97%)
Ni (tot)	11.9	2.46 (93%-97%)
Ni (sol)	8.9	0.20 (69%-97%)
V (tot)	5.7	0.26 (100%)
V (sol)	3.3	0.20 (97%-100%)
NO_3^- [a]	0.4	0.33 (100%)
SO_4^{2-} [a]	0.6	0.17 (100%)
OP^{AA}, OP^{GSH}, OP^{TOTAL}	<5	-

[a] measured in $PM_{2.5}$

"Tot" denotes total, while "sol" water-soluble metal extraction. "C" is the coarse and "F" is the fine PM fraction.

PM_{10}, $PM_{2.5}$, NO_3^- and SO_4^{2-} in μg/m^3, absorbance in 10^{-5}/m, trace metals in ng/m^3.

Precision in % coefficient of variation (CV).

Ranges in % > LOD for trace metals denote $PM_{2.5\text{-}10}$ and $PM_{2.5}$ fractions.

Table S4 Two-pollutant models of associations between air pollution exposure and percentage changes (post-pre) in FVC immediately after exposure (outdoor sites).

	IQR	ADJUSTMENT POLLUTANTS																									
		PM_{10}	$PM_{2.5}$	$PM_{2.5-10}$	PNC	Abs.[a]	EC (F)	EC (C)	OC (F)	OC (C)	Fe (tot)	Fe (sol)	Cu (tot)	Cu (sol)	Ni (tot)	Ni (sol)	V (tot)	V (sol)	End.	$NO_3^{-\,a}$	$SO_4^{2-\,a}$	OP^{AA}	OP^{GSH}	OP^{TOTAL}	O_3	NO_2	NO_X
**PM_{10}**	13.50	-0.14	-1.76	0.89	-0.22	-0.20	-0.18	0.07	0.05	-0.54	-0.09	-0.43	-0.10	-0.14	-0.13	-0.09	-0.09	-0.10	-0.18	-0.12	-0.11	-0.94	-0.34	-0.72	0.16	0.01	-0.11
**$PM_{2.5}$**	11.54	2.26	0.01	0.76	-0.24	-0.21	-0.17	0.25	0.17	-0.09	-0.03	-0.54	-0.05	-0.07	0.08	0.05	0.18	0.13	-0.03	0.74	0.14	-0.83	0.07	-0.02	0.39	0.17	-0.05
**$PM_{2.5-10}$**	8.23	-1.61	-1.07	-0.48	-0.37	-0.34	-0.34	-0.18	-0.11	-1.52**	-0.25	-0.57	-0.28	-0.36	-0.55	-0.37	-0.50	-0.48	-0.53	-0.52	-0.51	-1.19**	-0.93	-1.15*	-0.07	-0.24	-0.27
**PNC**	32,906	-1.38**	-1.41**	-1.29**	-1.34**	1.45	-0.21	-0.18	-1.22**	-1.41**	-2.33	-0.30	-1.43	-1.29	-1.34**	-1.06*	-1.38**	-1.39**	-1.39**	-1.64**	-1.50**	-1.50**	-1.31*	-1.51**	-0.53	-0.52	0.08
**Absorbance[a]**	3.49	-1.90**	-1.93**	-1.81**	-3.66	-1.87**	-5.85	-0.84	-1.83**	-2.03**	-3.65**	-1.05	-2.46	-2.71	-1.92**	-1.60**	-1.95**	-1.98**	-2.09**	-2.08**	-1.94**	-2.16**	-1.81**	-2.14**	-0.62	-0.82	0.07
**EC (F)**	4.35	-2.05**	-2.08**	-1.95**	-1.72	4.73	-2.03**	-0.20	-1.95**	-2.16**	-4.19	-0.74	-2.41	-2.37	-2.04**	-1.62*	-2.06**	-2.09**	-2.24**	-2.31**	-2.15**	-2.10**	-1.70*	-2.03**	-0.74	-0.65	0.57
**EC (C)**	0.40	-2.47**	-2.52**	-2.35**	-2.19	-1.51	-2.25	-2.44**	-2.22**	-2.43**	-2.86*	-1.52	-2.56	-2.70	-2.66**	-1.99**	-2.47**	-2.45**	-2.53**	-2.43**	-2.43**	-3.40**	-2.38**	-3.61**	-0.35	-1.09	-0.05
**OC (F)**	1.82	1.77**	1.78**	1.67**	1.54**	1.64**	1.63**	1.44**	1.73**	2.52**	1.50**	1.28*	1.37*	1.61**	1.67**	1.29	1.68**	1.69**	1.91**	1.73**	1.79**	2.02**	1.54**	1.80**	0.82	1.76**	1.43**
**OC (C)**	0.79	0.49	0.11	0.90*	-0.08	-0.16	-0.11	0.03	0.67*	0.07	0.05	-0.16	0.04	-0.04	-0.01	0.12	0.08	0.09	0.00	0.14	0.06	-0.54	-0.14	-0.46	0.06	-0.09	-0.12
**Fe (tot)**	895.10	-0.98**	-0.99**	-0.92*	0.85	1.21	1.25	0.27	-0.85*	-0.99**	-0.99**	0.19	0.79	-0.25	-0.94*	-0.70	-1.00**	-1.00**	-1.06**	-1.10**	-1.11**	-0.94*	-0.72	-0.88*	-0.48	-0.34	0.10
**Fe (sol)**	32.09	-2.29**	-2.39**	-2.07**	-1.68	-1.14	-1.49	-1.18	-1.71**	-2.12**	-2.27*	-2.02**	-2.19	-1.96	-1.96**	-1.64**	-2.07**	-2.08**	-2.19**	-2.75**	-2.40**	-2.26**	-2.06**	-2.21**	-1.17	-1.30	-0.90
**Cu (tot)**	57.96	-1.60**	-1.61**	-1.52**	0.06	0.57	0.29	0.12	-1.29*	-1.60**	-2.76	0.19	-1.61**	-0.94	-1.57**	-1.14	-1.59**	-1.60**	-1.76**	-1.77**	-1.80**	-1.54**	-1.23	-1.47*	-0.71	-0.68	-0.02
**Cu (sol)**	8.65	-1.50**	-1.51**	-1.43**	-0.12	0.78	0.25	0.24	-1.43**	-1.51**	-1.19	-0.07	-0.70	-1.50**	-1.44**	-1.12	-1.51**	-1.52**	-1.54**	-1.63**	-1.56**	-1.53**	-1.22	-1.47*	-0.61	-0.51	0.10
**Ni (tot)**	3.53	0.14	0.15	0.17	0.04	0.01	0.04	-0.08	0.12	0.15	0.07	0.08	0.03	0.07	0.14	0.15	0.13	0.14	0.13	0.14	0.13	-0.07	-0.12	-0.24	-0.13	0.05	0.03
**Ni (sol)**	1.82	-1.74**	-1.76**	-1.67**	-1.07	-1.11	-1.06	-0.94	-1.20	-1.78**	-1.26	-0.98	-1.11	-1.24	-1.77**	-1.76**	-2.08**	-2.73**	-1.73**	-1.83**	-1.94**	-2.13**	-1.93**	-2.07**	-1.02	-0.90	-0.54
**V (tot)**	2.04	-0.24	-0.33	-0.27	-0.24	-0.26	-0.19	0.05	-0.20	-0.27	-0.27	-0.34	-0.21	-0.26	-0.22	0.27	-0.27	-1.18	-0.25	-0.26	-0.23	-1.60	-0.69	-0.89	0.19	0.33	0.14
**V (sol)**	1.94	-0.19	-0.29	-0.20	-0.25	-0.33	-0.20	0.04	-0.08	-0.26	-0.27	-0.37	-0.19	-0.26	-0.22	0.84	1.19	-0.23	-0.23	-0.21	-0.19	-1.22	-0.73	-0.86	0.09	0.34	0.18
**Endotoxin**	0.19	0.01	0.00	0.01	0.00	-0.01	0.00	0.00	0.01	0.00	0.00	-0.01	0.00	0.00	0.00	0.00	0.00	0.00	0.00	0.00	0.00	0.00	0.01	0.00	0.00	0.00	-0.01
**$NO_3^{-\,a}$**	5.19	-0.04	-0.60	0.05	-0.46	-0.36	-0.37	-0.01	-0.03	-0.17	-0.29	-0.65*	-0.29	-0.27	-0.01	-0.19	-0.02	-0.06	-0.13	-0.12	0.00	-0.42	-0.19	-0.13	0.37	-0.03	-0.20
**$SO_4^{2-\,a}$**	2.99	-0.15	-0.21	-0.20	-0.34	-0.25	-0.26	0.00	-0.23	-0.14	-0.29	-0.44	-0.31	-0.21	-0.07	-0.29	-0.08	-0.12	-0.16	-0.17	-0.17	-0.14	-0.13	-0.08	0.38	0.00	-0.11
**OP^{AA}**	19.08	0.51	0.38	0.21	0.25	0.37	0.24	0.98*	-0.54	-0.05	0.12	-0.17	0.13	0.19	-0.22	-0.09	0.63	0.23	-0.09	0.22	-0.03	-0.11	0.25	1.57	0.71	0.82	0.47
**OP^{GSH}**	15.53	-0.66	-0.97	-0.36	-0.15	-0.18	-0.33	0.09	-0.49	-0.81	-0.49	-0.59	-0.46	-0.39	-1.05	-0.62	-0.78	-0.80	-1.18*	-0.88	-0.93	-1.07	-0.93	-1.27	-0.01	-0.27	-0.13
**OP^{TOTAL}**	38.71	0.19	-0.58	0.09	0.24	0.36	0.13	1.41	-0.73	-0.35	-0.10	-0.48	-0.07	0.03	-1.17	-0.41	-0.07	-0.28	-0.61	-0.48	-0.55	-2.67	0.51	-0.60	0.80	0.74	0.47
**O_3**	9.74	3.49**	3.55**	3.35**	3.00**	2.94**	3.00**	3.17**	3.02**	3.42**	3.12**	2.89**	3.07**	3.09**	3.73**	3.13**	3.56**	3.46**	3.42**	3.76**	3.90**	3.95**	3.34**	3.89**	3.38**	3.40**	2.82**
**NO_2**	10.54	-2.58**	-2.62**	-2.49**	-2.02*	-1.78	-2.06	-1.71	-2.66**	-2.68**	-2.26**	-1.89*	-2.15*	-2.21*	-2.58**	-2.21**	-3.01**	-2.88**	-2.77**	-2.57**	-2.58**	-3.54**	-2.46**	-3.24**	0.02	-2.58**	0.85
**NO_X**	28.05	-2.40**	-2.41**	-2.34**	-2.49**	-2.48*	-2.86**	-2.45	-2.24**	-2.54**	-2.61**	-1.90*	-2.47**	-2.57**	-2.46**	-2.22**	-2.58**	-2.56**	-2.62**	-2.45**	-2.40**	-2.71**	-2.33**	-2.68**	-0.65	-3.09	-2.41**

For explanation see Table S3.
